# Lyoplate-based multiparameter flow cytometry for the analysis of T cell subsets in human immuno-monitoring studies

**DOI:** 10.1186/1479-5876-9-S2-P18

**Published:** 2011-11-23

**Authors:** Federica Villanova, Paola Di Meglio, Susanne Heck, Margaret Inokuma, Ryan Brinkman, Esperanza Perucha, Maria Hernandez Fuentes, Graham Lord, Skip Maino, Frank O Nestle

**Affiliations:** 1 St. John’s Institute of Dermatology, King’s College London, London, UK; 2 NIHR GSTT/KCL Comprehensive Biomedical Research Centre, Guy's & St. Thomas' NHS Foundation Trust, London, UK; 3 Biological Research & Development, BD Biosciences, San Jose, CA, USA; 4 Terry Fox Laboratory, BC Cancer Agency, Vancouver, BC, Canada; 5 MRC Centre for Transplantation, King's College London, London, UK

## 

In recent years immuno-monitoring studies are becoming increasingly popular due to the relevant role the immune system plays in many pathologies and in treatment responses.

Human translational research is hampered by limited amounts of samples, intrinsic human variability and practical issues involving multi-centre sample collection and analysis. Therefore, human immuno-monitoring studies need to be standardized. Multicolour flow cytometry (MFC) provides a powerful tool to unravel the complexity of the immune system. However standardization of this technique is still in progress, due to differences in sample quality, reagents, antibodies and fluorchromes used, as well as instrument settings.

Part of this variability could be overcome by using lyophilized reagents in a 96 well plate format for cell stimulation and staining.

In this pilot study we assess how lyoplate based-MFC performs compared to traditional liquid reagent-based MFC, mirroring larger human immuno-monitoring cohorts.

Peripheral blood mononuclear cells were collected from healthy volunteers at two time points. Frozen samples were thawed, stimulated and stained using either liquid or lyophilized reagents. The 10 colour flow cytometry antibody cocktail used allowed the analysis of different T cell subsets (CD8 T cells, Th cells and Treg cells) and their cytokine production (IFNγ, IL17A, IL10). Quantitative and qualitative differences between liquid and lyophilized reagents were evaluated, as well as intra- and inter-assay variability.

Data from this study will assess the feasibility of standardized and high-throughput immuno-monitoring studies to discover pathology associated signatures and biomarkers predictive of therapy response.

